# Use of tactile feedback to control exploratory movements to characterize object compliance

**DOI:** 10.3389/fnbot.2012.00007

**Published:** 2012-07-26

**Authors:** Zhe Su, Jeremy A. Fishel, Tomonori Yamamoto, Gerald E. Loeb

**Affiliations:** ^1^Department of Biomedical Engineering, Medical Device Development Facility, University of Southern California Los Angeles, CA, USA; ^2^SynTouch LLC Los Angeles, CA, USA

**Keywords:** compliance discrimination, exploratory movements, haptic perception, Barrett robot, biomimetic tactile sensor (BioTac^®^), haptic robotics

## Abstract

Humans have been shown to be good at using active touch to perceive subtle differences in compliance. They tend to use highly stereotypical exploratory strategies, such as applying normal force to a surface. We developed similar exploratory and perceptual algorithms for a mechatronic robotic system (Barrett arm/hand system) equipped with liquid-filled, biomimetic tactile sensors (BioTac^®^ from SynTouch LLC). The distribution of force on the fingertip was measured by the electrical resistance of the conductive liquid trapped between the elastomeric skin and a cluster of four electrodes on the flat fingertip surface of the rigid core of the BioTac. These signals provided closed-loop control of exploratory movements, while the distribution of skin deformations, measured by more lateral electrodes and by the hydraulic pressure, were used to estimate material properties of objects. With this control algorithm, the robot plus tactile sensor was able to discriminate the relative compliance of various rubber samples.

## Introduction

Humans interact with compliant objects to judge ripeness of fruits, the air pressure in bicycle tires or the quality of a mattress. Expert bakers judge the quality of flour by evaluating physical firmness or toughness of dough (Katz, 1937). During breast or prostate examinations, healthcare practitioners use their hands to locate and characterize a hard lump in soft tissue. Unlike visual features such as size and shape, compliance can only be appreciated via active or passive touch.

It is essential for social and personal assistive robots and prosthetic hands (a form of telerobot) to be able to perceive material properties such as compliance to handle household objects. The ability to interact with fragile objects is necessary particularly if such systems are designed to interact physically with humans. Compliance perception could also be beneficial to robot-assisted, minimally invasive surgeries by detecting a hidden tumor in an organ or a calcified artery in heart tissue (Yamamoto et al., 2009). A variety of tactile sensors have been designed to solve the tactile sensing problems in robotic manipulation and medicine (Webster, 1988), but their practical use is limited by the hostile environments to which robotic and prosthetic hands are typically exposed. The BioTac^®^ is a robust and easy to repair tactile sensor that is capable of detecting point of contact, normal/tangential contact forces, and object spatial properties with impedance sensing electrodes (Wettels et al., 2008a; Wettels and Loeb, 2011), micro-vibrations associated with slip and textures through a hydro-acoustic pressure sensor (Fishel et al., 2008), and thermal fluxes with a thermistor (Lin et al., 2009).

Previous studies of compliance discrimination by robots used a combination of tactile and force sensors. (Takamuku et al., 2007) built a tendon-driven robot hand covered with strain gauges and a piezoelectric polyvinylidene fluoride (PVDF) skin. By performing squeezing and tapping over objects with different material properties, the strain gauges in this tactile sensor enabled the discrimination of hardness of different materials. Campos and Bajcsy (1991) proposed a robotic haptic system architecture that performed haptic exploratory procedures based on Lederman and Klatzky (1987) psychophysical studies of human performance. Hardness of objects were determined by measuring the force required to produce a given displacement. Both studies focused on measuring contact force and indentation displacement to discriminate object hardness or compliance. An adaptive force/position control algorithm was tested on an industrial robot to maintain force along the normal direction to the surface while moving in tangential directions on a rubber ball with 10 cm radius and 5000 N/m stiffness (Villani et al., 2000). In this paper, we present the results of using information about distributed deformation of the elastic skin of our tactile senor to discriminate compliance, a strategy that appears to be similar to that used by humans. This is made possible by using sensory feedback from a cluster of impedance sensing electrodes in the BioTac that are responsive to distributed forces. With these electrodes we were able to maintain a consistent orientation while applying normal forces to the surface of the object.

Subjective hardness/softness discrimination has been studied in psychophysical studies. Srinivasan and LaMotte (1995) showed that humans are efficient at discriminating subtle differences in softness under both active touch and passive touch with only cutaneous sensation but they are unable to discriminate even large differences during local cutaneous anesthesia. This suggests that tactile sensory information independent of proprioceptive information is necessary for discriminating softness of objects with deformable surface. Their studies also show that randomizing maximum force levels and indentation velocity in passive touch does not seem to affect sensitivity. This indicates that compliance discrimination can be done without fine control of these movements. Instead, we propose that spatial distribution of skin could be the cue for compliance discrimination. Peine (1999) developed a taxonomy that classifies the surgeons' finger motion during palpation procedures. They found that surgeons apply various normal force with no lateral motion to sense the stiffness of body tissues. Lateral motion after applying heavy pressure was found to enhance the ability to detect hard lumps in soft tissue.

To acquire information about object properties, humans tend to perform stereotyped exploratory movements Lederman and Klatzky, 1987. The exploratory movements to detect hardness are pressing and squeezing (Lederman and Klatzky, 1990). We have developed a haptic robot platform with a Barrett hand-wrist-arm system whose three fingers have been equipped with novel BioTac^®^ multimodal tactile sensors. In this paper, we present algorithms for the control of human-like exploratory movements for pressing on and characterizing objects with various hardnesses (durometer values). When robot gradually presses its fingertip into rubber samples with compliant surfaces, it uses the sensory feedback from the tactile sensor (BioTac) to control both normal and tangential contact forces and to adjust the orientation of its fingertip to account for the potentially unknown orientation of contact surfaces and internal discontinuities such as buried lumps. The distributed deformation sensed by the BioTac can be used to estimate the compliance of the contact surface.

## Materials and methods

We present data from initial experiments with flat objects made from materials with varying hardness to demonstrate the simultaneous use of multimodal tactile sensor data to control exploratory movements and to interpret their results.

### Experiment setup

#### Overview of the biomimetic tactile sensor (BioTac)

**Figure 1:**
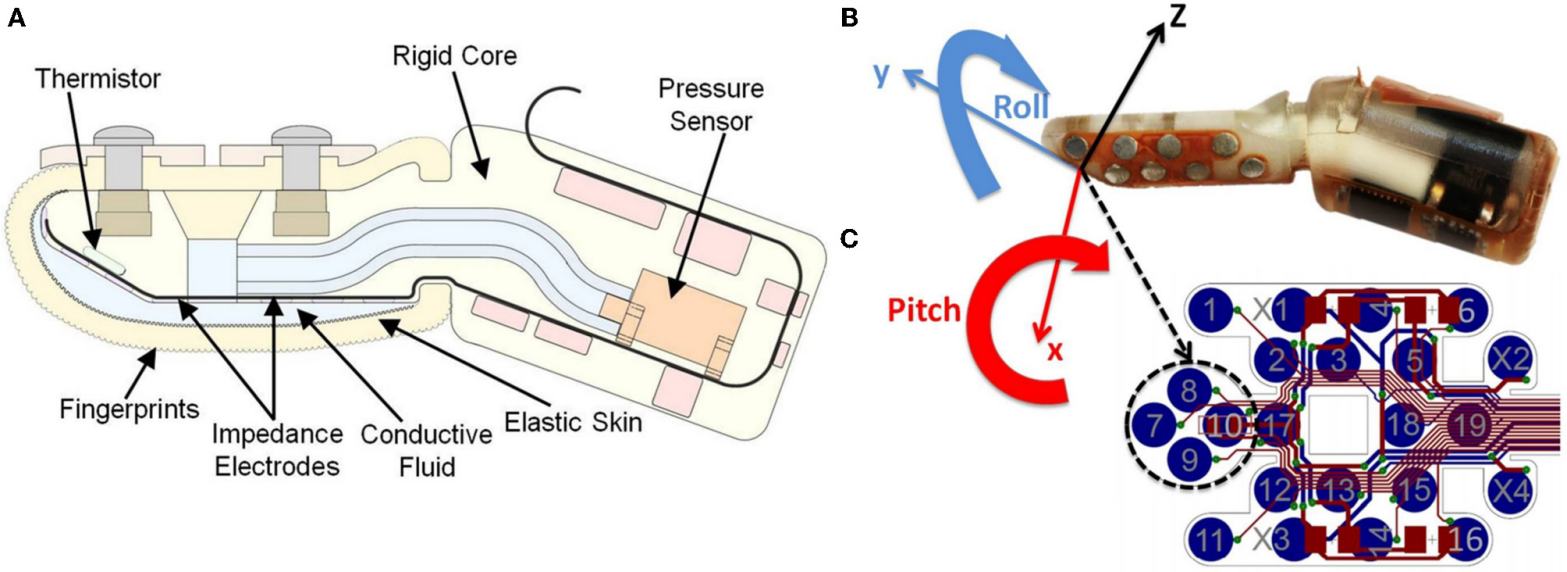
**(A)** Schematic diagram of the BioTac biomimetic tactile sensor. Sensing modalities include measurement of normal and shear forces detected by changes in impedance between electrodes as the conductive fluid pathways deform, slip-related microvibrations that propagate through the skin and fluid and are detected by the hydro-acoustic pressure sensor, and thermal properties as detected by a thermistor capable of detecting heat flow between the preheated core and contacted objects; **(B)** Orientations on BioTAC: the finger local coordinate frame has its origin in the center of the two electrode pairs and is coplanar with the flat surface of the core; **(C)** Electrode array map.

The BioTac (Figure 1A) consists of a rigid core housing all electronics and sensory components surrounded by an elastic skin that is inflated with an incompressible and conductive fluid. When the skin contacts an object, this fluid is displaced, resulting in distributed impedance changes in the electrode array on the surface of the rigid core. The impedance of each electrode tends to be dominated by the thickness of the fluid layer between the electrode and the immediately overlying skin. The skin has a pattern of asperities on its inner layer that gradually compress with increasing normal force, preventing object saturation (Wettels et al., 2008b). A MEMS pressure transducer measures hydrostatic pressure, which increases depending on the distribution of deformation in the elastic skin.

Similar to the human fingertip, the BioTac sensors are sensitive to tangential as well as normal forces. When performing a compliance movement it is desirable to apply forces normally and symmetrically to the object. For the haptic robot, this means servoing its end-effectors in the pitch and roll directions to orient a flat portion of the core of the BioTac that defines a local coordinate frame (Figure 1B). The sensory feedback is provided by four adjacent electrodes on this flat region whose impedance depends on compression of the skin against the electrode surface. These four adjacent electrodes are labeled electrode 7, 8, 9, and 10 on the electrode array map (Figure 1C). The pair of electrodes along the x-direction (8 and 9) and the pair of electrodes along the y-direction (7 and 10) are used for servocontrol of the pitch and roll, respectively, of the robotic fingertip. When the tactile sensor detects differences between these pairs of electrodes the error is corrected by adjusting the pitch or roll of the fingertip with the robot. The total contact force during indentation is estimated from the sum of impedance changes on all four electrodes. When pressing into a compliant object, the object has a tendency to wrap around the finger and the resulting forces can be measured by lateral electrodes not on the flat surface (such as 17). Comparing this change with the relative magnitude of impedance changes in the central four electrodes can yield substantial information about the compliance of the object. Additional information from the fluid pressure can also be used to characterize these changes. The sensor signals that provide information about compliance depend also on the curvature of the surface of the object, which must be estimated simultaneously from the complete temporal profiles of all sensor signals (Wettels and Loeb, 2011).

#### Testing materials

The levels of compliance for objects used in this experiment are classified by their durometers. The durometer is measured by the indentation depth into a material created by a given force on a standardized indenter with specific diameter. There are several scales of durometer depending on the diameter and configuration of the indenter, the spring forces applied on the tested materials. The samples in this experiment were all one inch thick and made from Neoprene rubber (50 Shore A) and polyurethane rubber (30 Shore A, 50 Shore 00, and 30 Shore OO), going from hard to soft.

#### Experimental procedure

**Figure 2:**
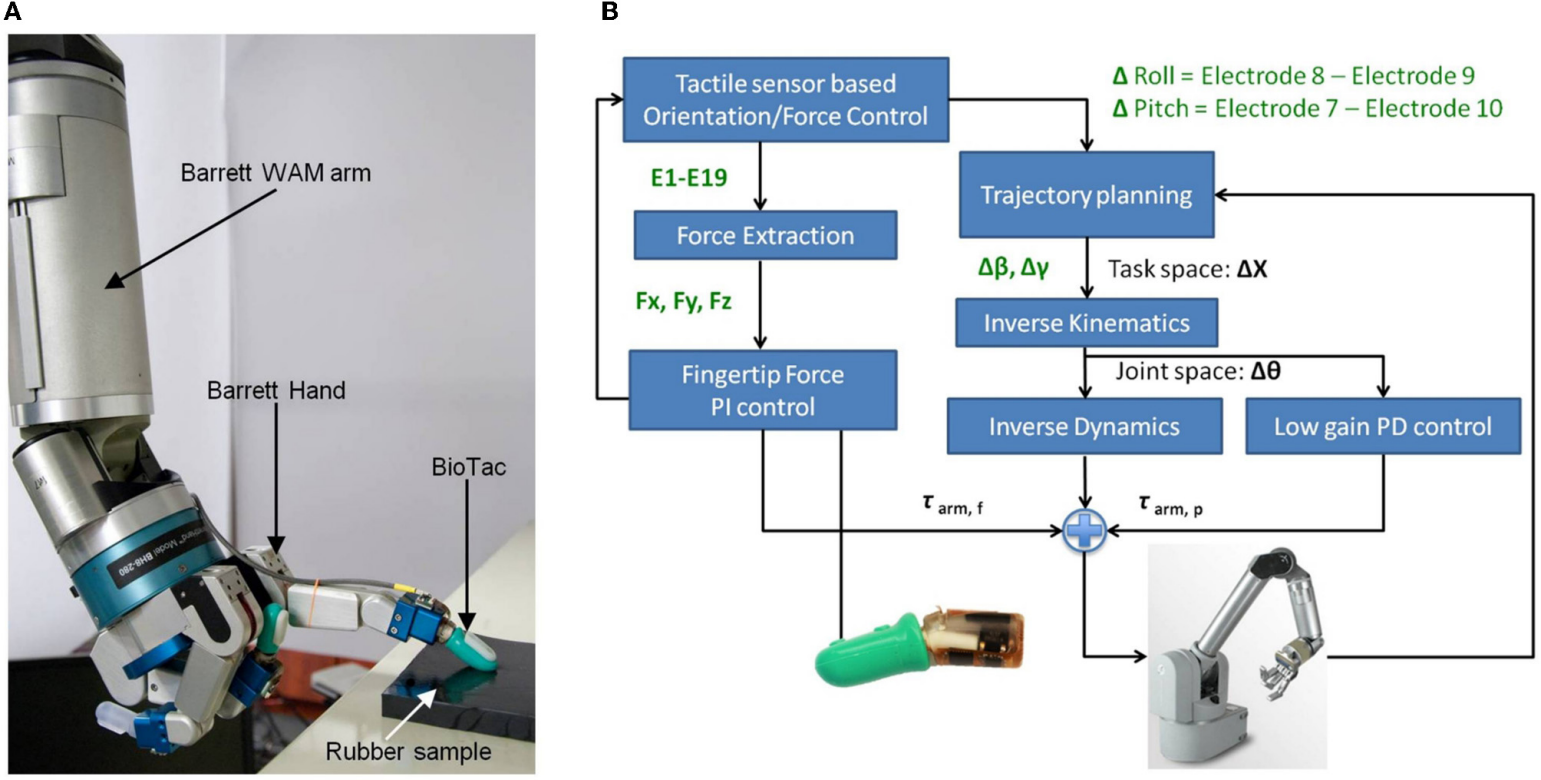
**(A)** Barrett with BioTac pressing a compliant surface; **(B)** Force/position control diagram.

The experiments were conducted on the seven DOF Barrett WAM robot arm and four DOF Barrett Hand BH-280 equipped with the BioTac. In each trial, the robot pressed one digit against a rubber sample in an unknown orientation and position. The robot controller had no prior knowledge of the orientation of the surface; instead it used tactile sensory feedback to identify a contact surface and adjust its finger orientation while pressing onto the compliant surface (Figure 2A).

### Robot exploratory movements

The exploratory movement can be divided into three phases: (1) Reach to an object surface by controlling position in Cartesian coordinate system with smooth path movement. The desired position is either provided *a priori* or estimated by machine vision. (2) Maintain normal contact and orientation with the center of the fingertip by maintaining a symmetrical distribution of force on a cluster of tactile sensors. (3) Controlling the exploratory movement, which consists of pressing the fingertip gradually into the contact surface while maintaining normal orientation of the fingertip in the pitch and roll directions.

#### Online orientation control using tactile sensor feedback

In order to maintain the orientation of the flat portion of the sensor while gradually pressing into a compliant surface, the desired orientation trajectory is generated by feedback signals on the two pairs of electrodes. These differential signals are used to incrementally increase or decrease current pitch and roll angles (β_*c*_, γ_*c*_) with very small increments (Δβ, Δγ) in the finger local coordinates, respectively. From the new local roll-pitch angles (β, γ) in the finger local coordinate frame (shown in Figure 1B), the corresponding finger local rotation matrix **R**_local_ can be derived and translated into rotation matrix in the robot base coordinates RBFfinger which is the forward kinematic from fingertip to robot base. Instead of using roll-pitch-yaw angles for orientation control directly, a unit quaternion representation of orientation [η,ϵ_1_,ϵ_2_,ϵ_3_] is derived from the new rotation matrix, because of its singularities-free property (Yuan, 1988). This online orientation generation algorithm is shown in a pseudo-code, (Table 1).

**Table 1:** **Algorithm for online orientation generation using tactile sensor feedback**.

Algorithm online orientation generation using tactile sensor feedback (*E*_8_, *E*_9_, *E*_7_, *E*_10_
IF E_8_ > E_9_ THEN
β = β_*c*_ + Δβ
*ELSEIF* E_8_<E_9_ THEN
β = β_*c*_ − Δβ
*ENDIF*
*IF* E_7_ > E_10_ THEN
γ = γ_*c*_ + Δγ
*ELSEIF* E_7_<E_10_ THEN
γ = γ_*c*_ − Δγ
*ENDIF*
**R**_local_ = **RotationMatrix** (β, γ)
${}_{F}^{B}{\text{R}}_{finger}{=}_{F}^{B}\text{R}*{\text{R}}_{local}$
[η, ϵ1, ϵ2, ϵ3]=Quaternions(RBFfinger)

The scalar η part and the vector part ϵ_1_,ϵ_2_,ϵ_3_) in a unit quaternion representation fulfill ${\text{η}}^{2}+\epsilon_{1}^{2}+\epsilon_{2}^{2}+\epsilon_{3}^{2}=1$.

A velocity-based orientation control with quaternion feedback is written below:

(1) $$\omega_{r}=\omega_{d}-K_{o}e_{o}$$

where ω_d_ is the desired angular velocities, **K**_o_ is diagonal gain matrix and **e**_o_ is the orientation error which is formulated using the unit quaternion (Yuan, 1988) as:

(2) $$e_{o}=\delta \epsilon =\eta_{d}\epsilon -\eta \epsilon_{d}+[\epsilon_{d}\times \epsilon ]$$

where $[\epsilon_{d}\times ]=\left[\begin{array}{ccc} 0 & -\epsilon_{\text{3}d} & \epsilon_{\text{2}d}\\ \epsilon_{\text{3}d} & 0 & -\epsilon_{\text{1}d}\\ -\epsilon_{\text{2}d} & \epsilon_{\text{1}d} & 0 \end{array}\right]$ and $\left[\text{η},\epsilon_{\text{1}},\epsilon_{\text{2}},\epsilon_{\text{3}}\right]$ is the current orientation and $\left[\text{η},\epsilon_{\text{1}d},\epsilon_{\text{2}d},\epsilon_{\text{3}d}\right]$ is the desired orientation.

#### Robot position control

The desired positions and orientation generated by the online trajectory generation using tactile sensory feedback is achieved by a velocity-based operational space controller together with an inverse dynamic law and PD feedback error compensation in joint space (Nakanishi et al., 2008). Inverse dynamics control enables low PD feedback gains for compliant control while ensuring high tracking performances. The control law is written as:

(3) $$\tau_{\text{arm},\text{ p}}=M{\ddot{q}}_{\text{d}}+h+K_{\text{p}}\left(q_{\text{d}}-q\right)+K_{\text{d}}\left({\dot{q}}_{\text{d}}-\dot{q}\right)$$

where τ_arm, p_ is computed vector of torques to track desired joint angles **q**_d_ with measured current joint angles **q**, **M** is rigid-body inertia matrix of the arm, ${\dot{\text{q}}}_{d}$ is the vector of desired joint velocity shown as:

(4) $${\dot{q}}_{d}=J^{+}({\dot{x}}_{d}+K_{x}(x_{d}-x))+K_{post}(I-J^{+}J)(q_{post}-q)$$

where x and x_d_ are the measured and desired finger position and orientation, **h** is the vector of Coriolis, centrifugal, and gravitational forces, **K**_p_, **K**_d_, **K**_x_, and **K**_post_ are diagonal gain matrices. **J** is the end-effector Jacobian, **J**^+^ denotes the pseudo-inverse of Jacobian and **q**_post_ is the vector of default posture optimized in the nullspace of the end-effector motion. The desired joint acceleration ${\overset{..}{\text{q}}}_{\text{d}}$ and desired joint position **q**_d_ are obtained by numerical differentiation and integration of the desired velocity ${\dot{\text{q}}}_{d}$.

#### Robot force control

Because the robot will press its end-effector onto compliant surfaces, external contact forces need to be taken into account. The external contact forces are obtained from the three force vectors on the BioTac extracted from impedance changes. They are used to compute torques in the joint space to account for the external contact forces by premultiplying them with Jacobian transpose, shown in Equation 5. The tracking of desired contact forces is achieved with a PI controller (Pastor et al., 2011)

(5) $$\tau_{arm,\;f}=-J^{T}(F_{arm\_des}-F_{arm})+K_{I}\overset{t}{\underset{t-\Delta t}{\int }}(F_{arm\_des}-F_{arm})dt$$

where **F**_arm_des_ is desired forces at the end-effector, **F**_arm_ is the measured forces interpreted from BioTac, **K**_I_ is a diagonal positive definite gain matrix and Δ*t* is the time-window during which the force error is integrated. The integral controller will compensate for steady-state errors during contact. An overview of the presented control architecture is shown in Figure 2B.

### Normal and tangential force extraction

During contact with an object, external forces deform the skin and fluid path around the impedance sensing electrodes. This deformation results in a distributed pattern of impedance changes on the electrodes. Previous studies have shown that both normal and tangential forces can be characterized from the impedance changes on the electrodes using machine learning techniques (Wettels et al., 2009; Wettels and Loeb, 2011). Here we present a simpler and more robust analytical algorithm to estimate normal and tangential forces.

**Figure 3:**
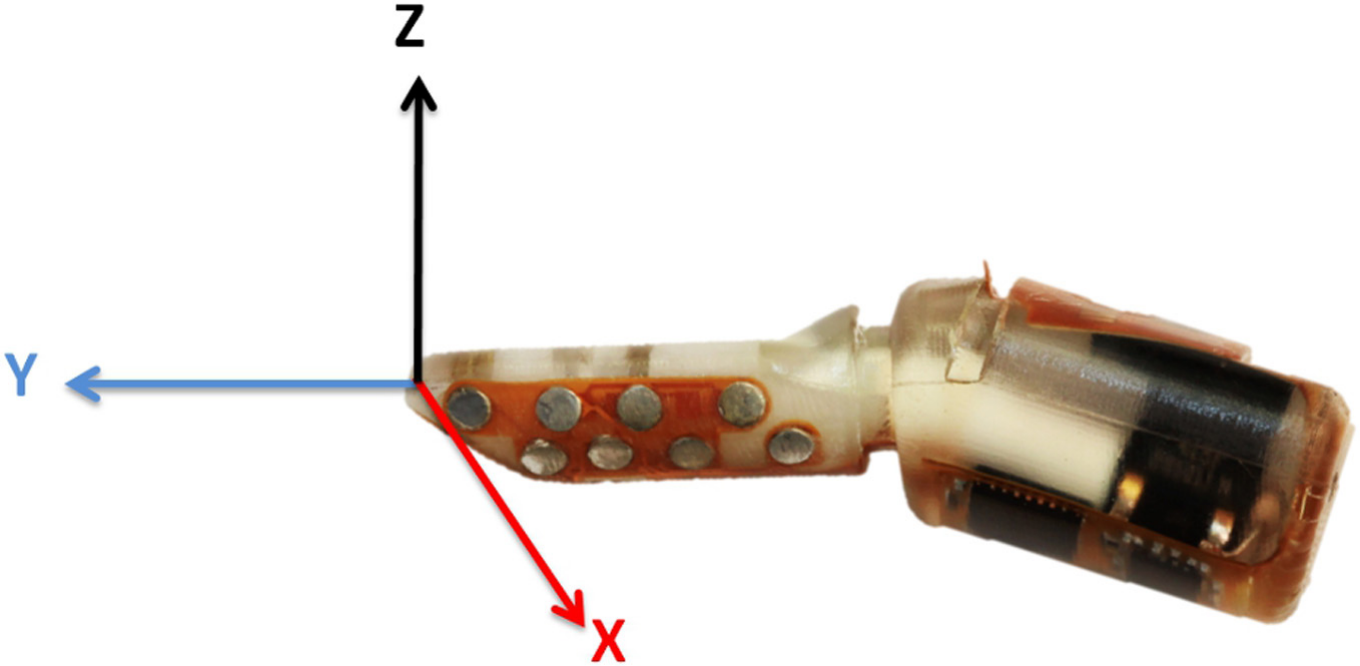
**Coordinate frame of BioTAC: each impedance sensing electrode has a specific orientation in the BioTac coordinate frame**.

The BioTac contains an array of 19 impedance sensing electrodes distributed over the surface of the core, which has a coordinate frame aligned with its long axis (Figure 3). Each impedance sensing electrode was determined to have the highest sensitivity to forces applied normally to its surface. The normal vectors to each of these electrodes in 3-axis coordinate space can be weighted with the change in impedance of these electrodes to determine an estimate of tri-axial force. We calculate the *x*, *y* and *z* force vectors from these electrodes with the following equation:

$$\left[\begin{array}{c} F_{x}\\ F_{y}\\ F_{z} \end{array}]=[\begin{array}{ccc} S_{x} & 0 & 0\\ 0 & S_{y} & 0\\ 0 & 0 & S_{z} \end{array}]\times [\begin{array}{ccc} N_{1,\;x} & \cdots & N_{19,\;x}\\ N_{1,\;y} & \cdots & N_{19,\;y}\\ N_{1,\;z} & \cdots & N_{19,\;z} \end{array}]\times ([\begin{array}{c} E_{1}\\ \cdots \\ E_{19} \end{array}]-[\begin{array}{c} E_{1,\;rest}\\ \cdots \\ E_{19,\;rest} \end{array}]\right)$$

where [F_x_, F_y_, F_z_] is the three dimensional force vectors on the BioTac, [E_1_ ⋯ E_19_] and [E_1, rest_ ⋯ E_19, rest_] are the measured impedance changes and resting impedance values on the BioTac, $\left[\begin{array}{ccc} {\text{N}}_{1,\;x} & \cdots & {\text{N}}_{19,\;x}\\ {\text{N}}_{1,\;y} & \cdots & {\text{N}}_{19,\;y}\\ {\text{N}}_{1,\;z} & \cdots & {\text{N}}_{19,\;z} \end{array}\right]$ is a matrix in which each column is calculated normal vector for each impedance sensing electrode surface from the geometry of the rigid core, the *S*_*x*_, *S*_*y*_ and *S*_*z*_ are scaling values for *x*-*y*-*z* three dimensional vectors to transform these arbitrary units into engineering units (N).

## Results

### Force extraction

**Figure 4:**
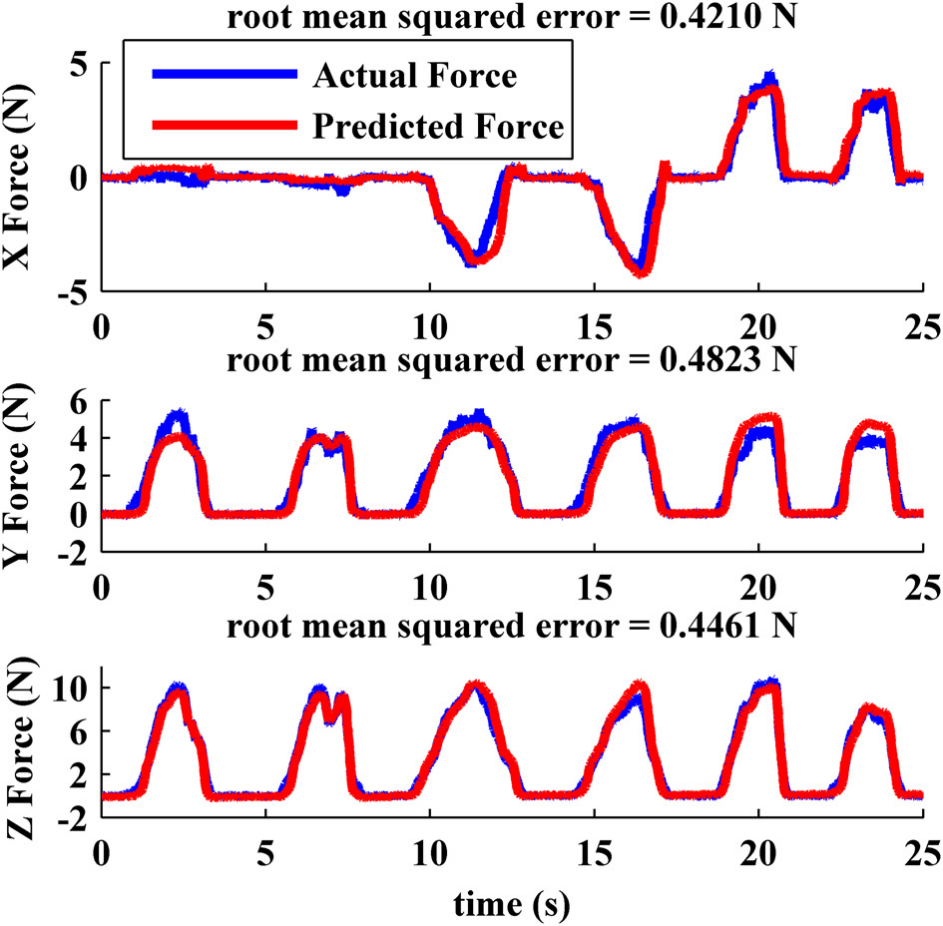
**Force measured on force plate (blue) and measured from the BioTac (red) for pokes with various tangential components**.

The scaling factor for each of the three dimensional estimated force vectors from the BioTac were calibrated on a 6-axis force plate (HE6x6-16, ATMI). We found that using the above-mentioned normal/tangential force calibration method was computationally efficient and achieved a low root-mean-squared (rms) error that exceeded performance of the neural network and machine learning techniques described in (Wettels et al., 2009; Wettels and Loeb, 2011). Figure 4 shows the actual forces (blue) measured from force plate and the predicted force vectors (red) extracted from BioTac by manually pressing and sliding the BioTac on the force plate. While pressing and sliding the BioTac, the flat portion of the BioTac was kept parallel with the surface of the force plate, similar to the orientation goal of the servocontroller for the exploratory poking movements. The rms errors for these sample movements were less than 10% of the applied forces in each axis.

### Pressing with orientation uncertainty

**Figure 5:**
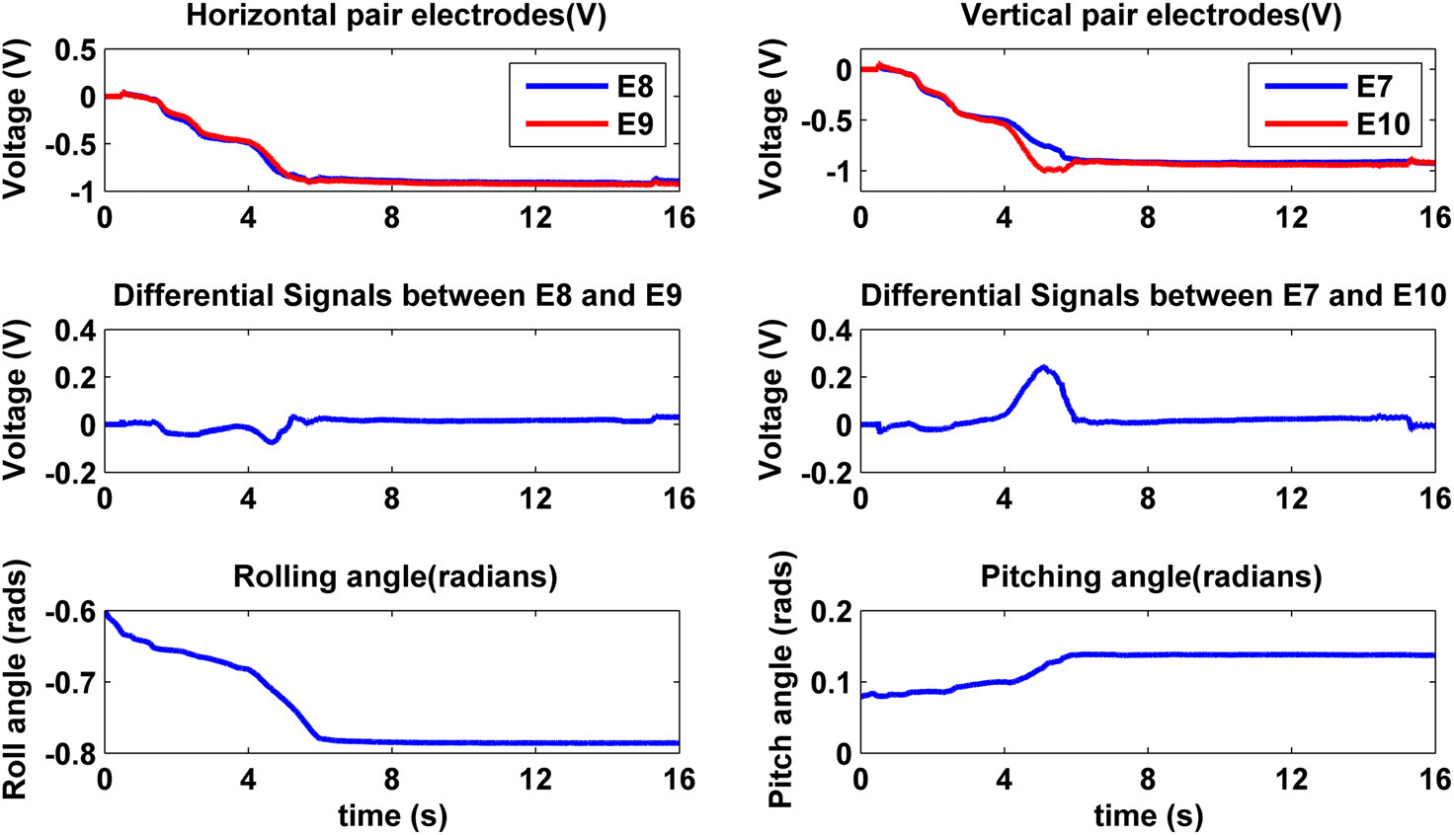
**Typical BioTac impedance sensor feedback on the point of contact and robot orientation behavior obtained from pressing on a compliant surface**.

Typical behavior of the system poking a surface with unknown orientation is illustrated in Figure 5. The top two plots show the impedances of the pairs of electrodes along x and y direction (E8 vs. E9, E7 vs. E10) on the flat portion of the core of the BioTac, which is the desired center of contact. The differential signals between those two pairs of electrodes are also displayed in the two middle plots in Figure 5. After the initial contact (around 0.5–1 s), the robot gradually pressed the BioTac into the compliant surface. A small asymmetry in the x-direction pair triggered the small correction to the roll angle that occurred at about 2–2.5 s and a larger correction at 4.5–5 s, shown in the bottom left plot. A larger asymmetry in the y-direction pair triggered a large pitch angle correction at 4–6 s, shown in the bottom right plot. The correction to pitch angle was relatively slow because it involved most of the proximal joints of the Barrett arm and it actually resulted in the second correction to the roll angle.

**Figure 6:**
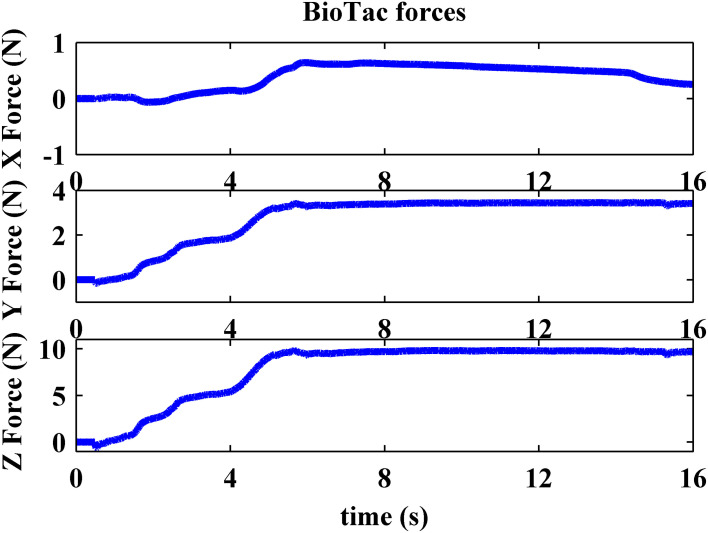
**Typical BioTac tangential force (X force) and normal force (Z force) feedback obtained from pushing on a compliant surface**.

While the robot performed a pressing behavior, its contact force on the compliant surface was controlled by using tangential and normal force feedback extracted from the impedance electrode array on the BioTac. Figure 6 shows that the robot pressed 10 N on the compliant surface and kept its lateral tangential force (X Force) close to zero. The axial tangential force of 4 N is what would be expected given the 30° tilt of the flat portion of the fingertip with respect to the long axis of the BioTac. Stretch between the BioTac elastic skin and the compliant rubber sample caused by two rolling movements (around 2–2.5 s and 4.5–5 s in Figure 5) created a positive tangential force on the sensor, but the force controller gradually decreased the tangential force to close to zero by the end of the movements shown in Figure 6.

### Compliance discrimination

#### Force and displacement

**Figure 7:**
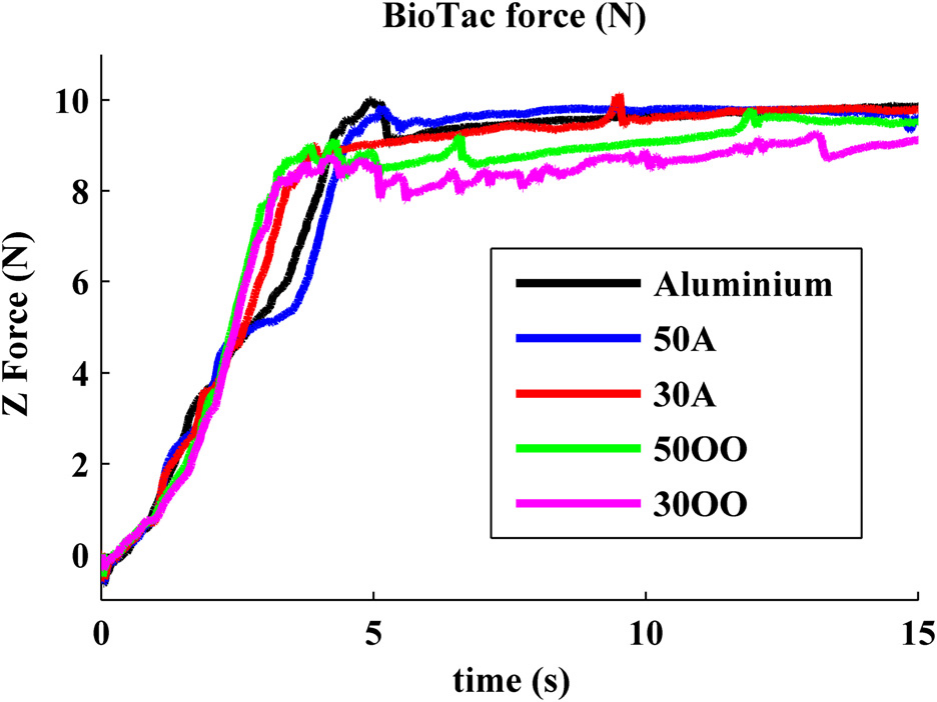
**Measured normal force from BioTac: five objects with five different hardness are tested with Barrett robot equipped with BioTac**.

**Figure 8:**
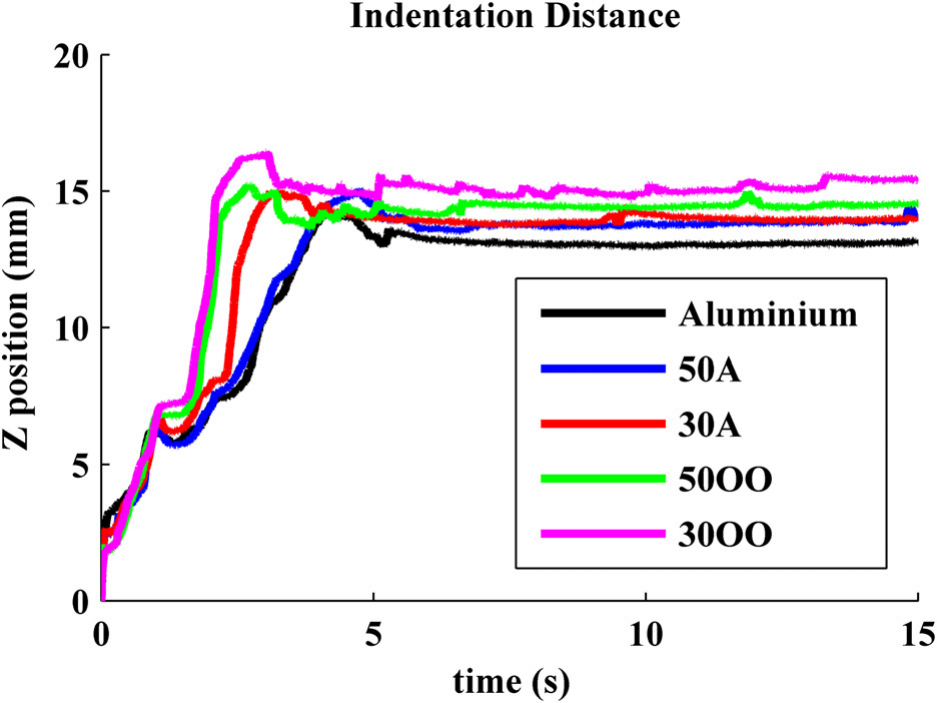
**Measured indentation displacement from Barrett joint encoder**.

**Figure 9:**
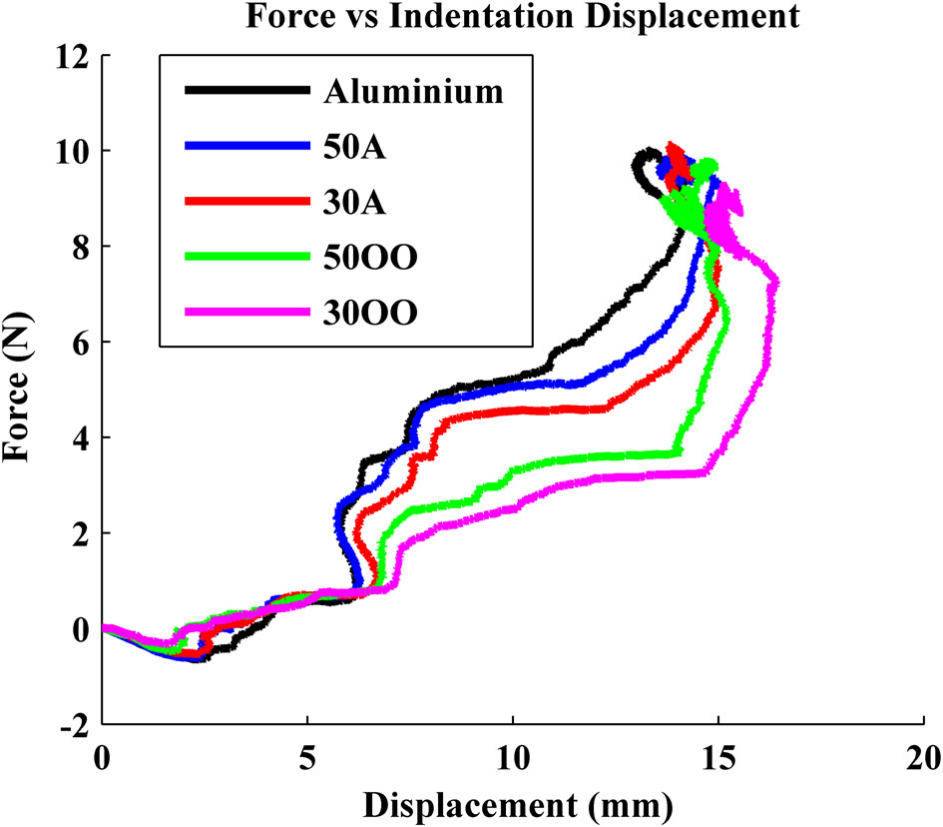
**Force vs. indentation displacement**.

Previous experiments showed that force and indentation displacement can be used in compliance discrimination when actively palpating with a tool (LaMotte, 2000). Thus, the ratio between force and indentation displacement can also provide information for the perception of compliance, especially for compliant objects covered with non-deformable surfaces, such as piano keys. During our experiment, the robot is controlled to apply 10 N in the normal direction onto five objects, consisting of an aluminum plate and four progressively softer rubber samples with durometer Shore 50A, Shore 30A, Shore 50OO, and Shore 30OO. Shore 50A is as hard as a pencil eraser and shore 30OO is a little bit softer than a racquet ball. When the robot was actively pressing the BioTac onto five objects with various hardnesses, normal forces and indentation displacement were measured from BioTac and robot joint encoders, respectively, as shown in Figures 7 and 8. The initial rates of rise of force were similar for all materials, but it took the robot longer to reach 10 N on soft materials 50OO and 30OO because it needed to constantly adjust its fingertip orientation to keep its fingertip orthogonal to the surface of soft materials. In Figure 8, we observe that the softer materials have more indentation displacement than the harder materials. The indentation displacement was measured by the position sensors in the robot actuators. It reflects the sum of indentation of the skin of the BioTac plus indentation of the object being probed plus stretching of the fine stainless-steel cables that link the motors to the joints. Figure 9 shows that indentation displacement trajectories were similar for all materials up to about 6 mm and 1 N, which were due to displacement of the elastic skin on the BioTac and the initial stretching on the finger joints, which explain a nearly linear relationship between force and displacement. From 6 mm to 12 mm, indentation displacement trajectories diverged as a result of the deformation of rubber samples with different compliance properties and stretching of cables in the robot. At about 15 mm and 10 N, they reconverged because they were then dominated by the stretching of the cables in the robot wrist subjected to large external forces from the compressed samples.

#### Deformation

In light of the complex combination of factors that contributes to apparent indentation displacement of the fingertip, it would be desirable to use temporal variations of average pressure and spatio-temporal variations of distributed skin deformation, as proposed for human discrimination of hardness by Srinivasan and LaMotte (1995). Both types of tactile information are available from the BioTac. The MEMS pressure transducer measures the average pressure of the fluid inside the space between the elastic skin and rigid core. The spatio-temporal variations of distributed deformation are provided by the impedance electrode array, especially from lateral electrodes adjacent to the central four electrodes used for controlling the applied force.

**Figure 10:**
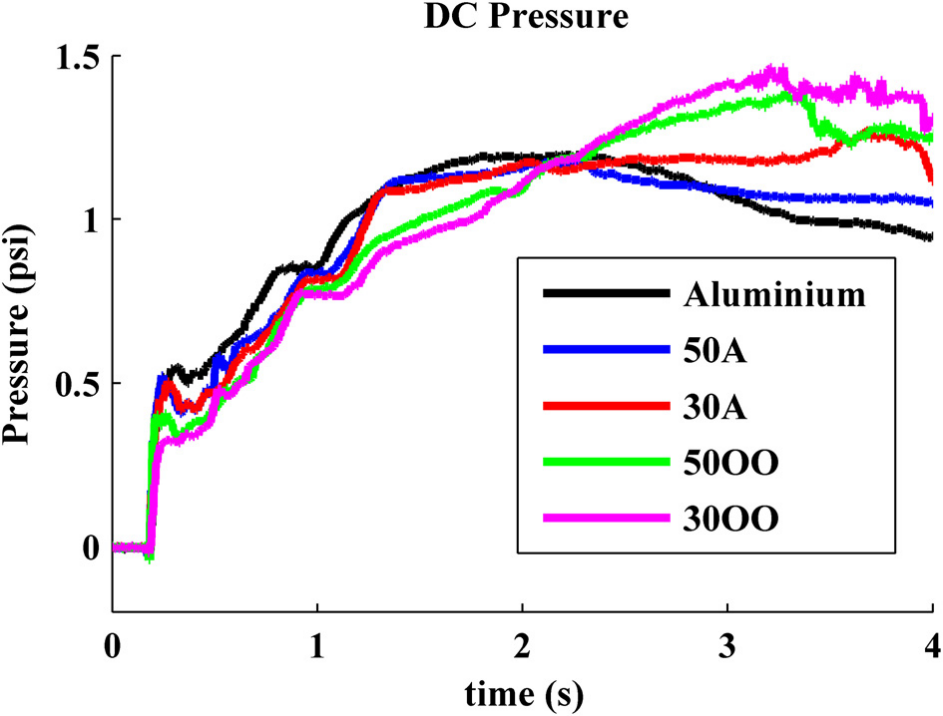
**Measured average pressure from MEMS pressure transducer on BioTac: the rate of average pressure and saturation pressure are used to discriminate object compliance**.

When the BioTac was pressed against hard materials (e.g., the aluminum plate and shore A 50 and shore A 30 rubber samples), fluid pressure plateaued or actually declined after normal force reached 2.87 N (around 2 s in Figure 10). This saturation is caused by the rigid object pushing the elastic skin against the rigid core on BioTac. The first part of the increasing fluid pressure reflects the compliance of the BioTac skin and fluid pressure, which grows nonlinearly after the skin contacts the core. The curves diverge before that occurs if and when the object compliance exceeds the BioTac compliance. As shown in Figure 9 (1–2 s), harder objects created a higher rate of average pressure changes in the BioTac. When BioTac pressed objects with softer surface, the soft surface not only pushed the elastic skin against the rigid core more gradually, but also progressively enveloped the side of the BioTac fingertip. This created higher saturation pressures for softer surfaces (around 2–3 s in Figure 10).

**Figure 11:**
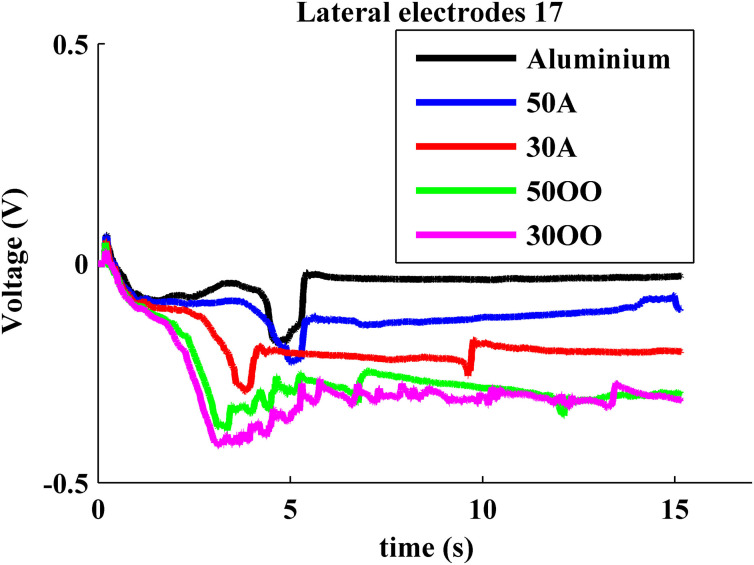
**Typical BioTac lateral impedance electrode feedback from pressing on different compliant surfaces**.

The tendency of soft surfaces to envelop the fingertip as they are deformed can be seen also in the impedances of more lateral electrodes such as #17 (Figure 11). The BioTac actually measures the current admitted into the electrode from a test pulse applied to various reference electrodes distributed in the fingertip, so a decrease in measured voltage from an initial value reflects an increase in electrode impedance. For the lateral electrode #17, the impedance initially increased similarly for all materials as increasing force was applied at the fingertip and the skin deformed, but the curves diverged as the more compliant materials deformed and enveloped the skin further from the centroid of contact. The reorientation movement that the robot made to correct pitch to maintain normal force (4.5–5 s in Figure 5) resulted in the transients in lateral electrode impedance at that time (Figure 11), which were particularly pronounced for the hard materials. After the robot corrected its orientation and reached its maximum contact force, the resting voltage on the lateral electrode reflected the compliance of the object.

## Discussion

The tactile sensors available in the BioTac have properties similar to those in human fingertips and can be used to measure compliance of objects, but only if there is accurate control of the exploratory movement. Those same sensors can be used to control the exploratory movements, using tactile feedback control that may also be similar to what humans use when deciding how to palpate an unknown object. The preliminary results presented here are a first step in designing algorithms that can enable robots to produce the range of exploratory movements and the percepts that humans achieve thereby.

In this paper, the BioTac was controlled to explore flat compliant objects. Compliant objects that have curved surfaces or inhomogeneities in material properties will generate different responses in the sensors, whose interpretation may require additional exploratory movements. The tactile-based control of exploratory movements presented here should enable systematic exploration of such unknown objects regardless of their location or orientation with respect to the robot hand.

Systematic datasets need to be generated by poking the BioTac into objects with various curvatures and various compliances to develop a more complete perceptual algorithm. In previous studies, the impedance sensing electrodes of the BioTac could be used to make coarse determinations of radius of curvature of rigid objects (Wettels and Loeb, 2011). Humans tend to follow the contour of objects to perceive their precise shapes (Lederman and Klatzky, 1990). Palpation of hard objects buried in soft tissues probably reflects a combination of tactile-driven movements to determine the orientation of hard surfaces and kinesthesia to keep track of the location and size of those surfaces (Peine, 1999). In the future, we will combine pressing and contour-following exploratory movements to facilitate the perception of both compliance and shape of objects. Eventually, tactile information from exploratory movements must be fused with machine vision to permit location, characterization, identification, and dexterous manipulation of objects in the environment.

### Conflict of interest statement

Jeremy A. Fishel and Gerald E. Loeb are equity partners in SynTouch, LLC, which manufactures and sells the BioTac sensors described in this article. Zhe Su and Tomonori Yamamoto have no commercial or financial relationships that could be construed as a potential conflict of interest.

## References

[B1] Campos M. Bajcsy R. (1991). “A robotic haptic system architecture,” in Proceedings of the 1991 IEEE International Conference on Robotics and Automation, (Sacramento, CA), 338–343.

[B2] Fishel J. A. Santos V. J. Loeb G. E. (2008). “A robust micro-vibration sensor for biomimetic fingertips,” in Proceedings of the IEEE International Conference on Biomedical Robotics and Biomechatronics, (Scottsdale, AZ), 659–663. 10.1109/BIOROB.2008.4762872

[B3] Katz D. (1937). Studies on test baking: III. The human factor in test baking: a psychological study. Cereal Chem. 14, 382–396.

[B4] LaMotte R. H. (2000). Softness discrimination with a tool. J. Neurophysiol. 83, 1777–1786. 10758090 10.1152/jn.2000.83.4.1777

[B5] Lederman S. J. Klatzky R. L. (1987). Hand movements: a window into haptic object recognition. Cogn. Psychol. 19, 342–368. 10.1016/0010-0285(87)90008-9 3608405

[B6] Lederman S. J. Klatzky R. L. (1990). “Haptic exploration and object representation,” in Vision and Action: The Control of Grasping, ed M. Goodale (New Jersey, NJ: Ablex), 98–109.

[B7] Lin C. H. Erickson T. W. Fishel J. A. Wettels N. Loeb G. E. (2009). “Signal processing and fabrication of a biomimetic tactile sensor array with thermal, force and microvibration modalities,” in Proceedings of the IEEE International Conference on Robotics and Biomimetics, (Guilin), 129–134.

[B8] Nakanishi J. Cory R. Mistry M. Peters J. Schaal S. (2008). Operational space control: a theoretical and emprical comparison. Int. J. Rob. Res. 27, 737–757.

[B9] Pastor P. Righetti L. Kalakrishnan M. Schaal S. (2011). “Online movement adaptation based on previous sensor experiences,” in Proceedings of the IEEE/RSJ International Conference on Intelligent Robots and Systems (IROS), (San Francisco, CA, USA). 10.1109/IROS.2011.6048160

[B10] Peine W. J. (1999). Remote Palpation Instruments for Minimally Invasive Surgery. Ph.D. dissertation, Harvard University, United States. (Dissertations and Theses: A&I database, publication No. AAT 9921526.).

[B11] Srinivasan M. A. LaMotte R. H. (1995). Tactual discrimination of softness. J. Neurophysiol. 73, 88–101. 7714593 10.1152/jn.1995.73.1.88

[B12] Takamuku S. Gómez G. Hosoda K. Pfeifer E. (2007). “Haptic discrimination of material properties by a robotic hand,” in Proceedings of the 6th IEEE International Conference on Development and Learning, (London), 1–6.

[B13] Villani L. Natale C. Siciliano B. de Wit C. C. (2000). An experimental study of adaptive force/position control algorithms for an industrial robot. IEEE Trans. Control Syst. Technol. 8, 777–786.

[B14] Webster J. G. (1988). Tactile Sensors for Robotics and Medicine. New York, NY: John Wiley and Sons.

[B15] Wettels N. Fishel J. A. Su Z. Lin C. H. Loeb G. E. (2009) “Multi-modal synergistic tactile sensing,” in Tactile Sensing in Humanoids—Tactile Sensors and Beyond Workshop, 9th IEEE-RAS International Conference on Humanoid Robots. (Paris).

[B16] Wettels N. Loeb G. E. (2011). “Haptic feature extraction from a biomimetic tactile sensor: force, contact location and curvature,” in IEEE-ROBIO, (Phuket, Thailand).

[B17] Wettels N. Santos V. J. Johansson R. S. Loeb G. E. (2008a). Biomimetic tactile sensor array. Adv. Robot. 22, 829–849.

[B18] Wettels N. Smith L. M. Santos V. J. Loeb G. E. (2008b). “Deformable skin design to enhance response of a biomimetic tactile sensor,” in IEEE International Conference on Biomedical Robotics and Biomechatronics, (Scottsdale, AZ), 132–137.

[B19] Yamamoto T. Vagvolgyi B. Balaji K. Whitcomb L. L. Okamura A. M. (2009). “Tissue property estimation and graphical display for teleoperated robot-assisted surgery,” in IEEE Transactions on Haptics, (Kobe), 4239–4245.

[B20] Yuan J. (1988). Closed-loop manipulator control using quaternion feedback. IEEE J. Rob. Autom. 4, 434–440.

